# Revision Hip Arthroplasty Through a Gluteal-Sparing Extended Posterior Approach May be Able to Achieve Similar Functional Outcomes to Primary Hip Arthroplasty

**DOI:** 10.1016/j.artd.2025.101681

**Published:** 2025-04-12

**Authors:** Dominic Thewlis, Jasvir Bahl, Hao Wei (Harvey) Chai, Stuart A. Callary, Thomas M. Grace, John B. Arnold, Mark Taylor, Lucian B. Solomn

**Affiliations:** a Centre for Orthopaedic and Trauma Research, Adelaide Medical School, University of Adelaide, Adelaide, Australia; b Department of Orthopaedics and Trauma, Royal Adelaide Hospital, Adelaide, Australia; c Alliance for Research in Exercise, Nutrition and Activity (ARENA), UniSA Allied Health and Human Performance, University of South Australia, Adelaide, Australia; d College of Science and Engineering, Medical Device Research Institute, Flinders University, Adelaide, Australia

**Keywords:** Gait analysis, Patient-reported outcomes, Hip replacement, Hip abductors

## Abstract

**Background:**

Revision total hip arthroplasty (THA) has been reported to have worse outcomes when compared to primary procedures, which may, in part, be due to the increased exposure required for the procedure. We aimed to investigate the postoperative functional outcomes of 2 groups of primary and revision THA, when revision procedures were performed using a gluteal-sparing extended posterior approach.

**Methods:**

Two groups of 51 primary and 21 revision THAs were prospectively recruited from a single center between 2016 and 2019. Both groups were assessed preoperatively using quantitative gait analysis and patient-reported outcomes, and at 3 and 12 months postoperatively. Hip and knee kinematics were computed from motion capture data acquired at the gait analysis. Kinematic and patient-reported outcome measures data were analyzed using linear mixed models. Statistical parametric analysis complemented the main analysis of the kinematics.

**Results:**

Patients in the primary group had worse preoperative patient-reported outcome measures when compared to the revision group. There were no between-group differences in walking speed. Hip extension in late stance phase of gait was 9° and 5° lower for the revision group when compared to the primary group at 3 and 12 months, respectively. These differences were not statistically significant, but the magnitude of the effect size was noteworthy suggesting a functional deficit (Cohen’s *d* = 0.64 and 0.54, respectively).

**Conclusions:**

Revision THA using a gluteal-sparing extended posterior approach may be able to achieve similar patient-reported and gait outcomes with those of primary THA within the first 12 postoperative months.

## Introduction

Primary total hip arthroplasty (THA) is a reliable and successful surgical procedure to relieve pain and improve self-reported functional outcomes in patients with end-stage hip osteoarthritis (OA) [1,2]. While deficits in gait remain relative to healthy individuals, and real-world physical activity remains low up to 2 years postoperatively [3], most patients report satisfaction with the procedure [4]. Unfortunately, many primary THA patients continue to require revision THA [5,6], which is typically associated with a higher complication rate and lower patient satisfaction compared to primary THA [5,7]. Registry data [8] show that the survivorship of revision THA is much poorer than that of primary THA and the 10-year survivorship of a first-time revision THA is 64% compared to 95% for primary THA [8]. Subsequent second and third rerevision THA surgeries are known to have even worse outcomes and survivorship [9]. Comparative studies report that revision THA has poorer patient-reported outcome measures (PROMs) in terms of both pain and function compared with primary THA [[10], [11], [12], [13]]. To our knowledge, no study has compared the functional outcome of patients following primary and revision THA using objective techniques, such as instrumented gait analysis. Without an objective analysis of function, it is challenging to accurately define functional targets to inform patient expectations following revision THA.

The most common aseptic reasons for revision THA are implant loosening and dislocation [14], both of which can directly affect patients’ symptoms and function. The need for increased exposure in revision surgery can cause abductor muscle damage [15], exacerbated by re-revisions [16], which is recognized to cause pain, dysfunction, and instability. Gluteal muscle damage is also known to increase loading of the hip joint [17], and can, therefore, influence acetabular component stability and osseointegration. The use of a gluteal-sparing extended posterior approach (Adelaide Extended Approach) was shown to protect the abductors, specifically the glutei, and significantly improve abductor function after revision THA for severe, Paprosky III acetabular defects. To improve the outcomes of our revision THA, we introduced the Adelaide Extended Approach in our practice.

The aim of this preliminary prospective study was to assess whether preservation of the abductors at revision THA can lead to PROMs and objective gait function comparable to those of primary THA. Our specific research questions were: (1) do PROMs differ between patients with a primary and revision THA preoperatively and up to 12 months postoperative; (2) do walking gait kinematics differ between patients with a primary and revision THA preoperatively and up to 12 months postoperative; and (3) do gait outcomes differ between single-stage and multistage revision THA. Based on the current evidence, it was reasonable to hypothesize that patients with revision THA would self-report worse outcomes and demonstrate reduced gait function compared to primary THA. However, the gluteal-sparing surgical approach used here aims to reduce risk factors associated with poorer functional outcomes. Therefore, we hypothesized that revision THA, when performed through the Adelaide extended posterior approach, would result in comparable outcomes to their peers with primary THA in the absence of major comorbidities and preoperative abductor function deficiency.

## Material and methods

Patients scheduled to undergo primary and revision THA at a public hospital in South Australia between November 2016 and November 2019 were recruited prospectively to this study and followed up to 1 year postoperative (3 time points: preoperative, 3 months, and 1 year postoperative). The study protocol was approved by our Human Research Ethics Committee (protocol number: R20160807). All participants provided written informed consent prior to being enrolled within the study. Patients for the primary THA group were eligible if they were undergoing an elective procedure for OA. Patients for the revision THA group were eligible if they were scheduled for revision surgery. Both groups had the same exclusion criteria: (1) concurrent neurological or neurodegenerative conditions; (2) concurrent pregnancy; (3) inability to understand written or spoken English; and (4) inability to walk unassisted for 10 meters during the gait assessment.

### Surgical technique and implants

Primary THAs were performed through a posterior approach and all participants received the same implant design: a press-fit Trilogy acetabular component and a cemented collarless polished tapered stem femoral stem (Zimmer Biomet, Warsaw, Indiana). Revision THAs were performed through an Adelaide extended posterior approach [15]. Briefly, with the patient in lateral decubitus, the skin is incised along the femur to the level of the greater trochanter and then along a line that connects the tip of the greater trochanter to the midpoint between the posterior superior iliac spine and the iliac tuberosity, which marks the anterior expansion of the insertion of gluteus maximus on the iliac crest. Fascial incision is performed in line with the skin incision over the femur and the anterior border of gluteus maximus in the gluteal region, marked by the perforating branches of the superior gluteal bundle. This allows preservation of the entire gluteus maximus as it is reflected posteriorly on its neurovascular pedicles as well as the exposure of the posterior margin of the gluteus medius along its insertion on the posterior gluteal line of the ilium, above the sciatic notch. During deep dissection, the Gluteus medius is mobilized anteriorly from the posterior gluteal line, above the sciatic notch, and the anterior division of the superior gluteal neurovascular bundle is exposed between gluteus medius and minimus. With the bundle under direct vision, the gluteus medius and minimus are mobilized anteriorly until penetrated by the ramifications of the superior gluteal nerve, about 2–3 cm anterior to the sciatic notch. This allows direct vision and controlled tension of the superior gluteal bundle during the surgery, and implicitly its protection during the acetabular reconstruction, even when the acetabular defect extends proximal to this bundle. The use of a nerve stimulator, we use a Checkpoint nerve stimulator (Checkpoint Surgical, Inc. Independence, OH), helps ensuring the preservation of the innervation to gluteus medius and minimus at the end of the procedure. When acetabular components were revised, they were revised to either (1) a Trilogy acetabular component when press fit and more than 50% implant to host bone contact was deemed achievable or (2) a Trabecular Metal modular/Trabecular Metal Acetabular Revision System acetabular component (Zimmer Biomet) when press fit was not possible or the implant to host bone contact was deemed to be less than 50%. Fixation of both Trilogy and Trabecular Metal modular components was augmented with 2–3 screws. If the Trabecular Metal Acetabular Revision System was used, the acetabular component was built and fixation achieved as described by Bunting et al [18]. When the stem was revised, either a collarless polished tapered stem or Zimmer Modular Revision [19] stem (Zimmer Biomet) was implanted. For this cohort, stems were cemented and performed through a cement-within-cement exchange [20], impaction bone grafting [18], or by cementing alone of a long stem [21].

### Patient follow-up, demographics, and outcome scores

All patients were reviewed preoperatively and followed up as per our departmental protocol, with clinical and radiographic reviews at 3 months and 1 year after surgery. Demographics as well as the Charlson Comorbidity Index were recorded preoperatively for each patient. At each review time point, participants completed a Hip Dysfunction and Osteoarthritis Outcome Score (HOOS) and underwent a quantitative 3-dimensional gait analysis. The sport and recreation domain of the HOOS domain was excluded as patients with end-stage OA and post-THA do not report adequate participation levels to yield meaningful scores. Complications that could have influenced hip outcomes were documented.

### Gait analysis and musculoskeletal modeling

Retroreflective markers (⌀ 14 mm) were placed atop anatomical landmarks of the pelvis and lower limbs [22,23]. A 10-camera motion analysis system (Vicon, Oxford, UK) recorded marker trajectories (100 Hz) while patients walked at self-selected speeds. A minimum of 3 walking trials per session were collected. A generic lower body musculoskeletal model (Gait 2392) was scaled using the Musculoskeletal Atlas Project Client (MAPClient) [24,25]. Skin-surface markers were used to perform a principal component morphing of the MAPClient pelvis, thigh, and lower leg. The trunk and feet were linearly scaled in OpenSim as statistical shape models for these segments are not currently implemented in the MAPClient. Lower limb joint kinematics were computed in OpenSim [26] according to the convention recommended by the International Society of Biomechanics [27,28]. Spatiotemporal and kinematic variables for the pelvis, hip, and knee were extracted for analysis. The coronal plane kinematics of the pelvis were used to categorize the presence of a positive Trendelenburg gait or Duchene’s gait, respectively [29,30].

### Data analyses

Group characteristics were compared by time point (group∗time), using univariate analysis of variance or Fisher’s exact tests. Linear effects mixed models were used to test the between-group differences in self-reported outcomes and gait function at each time point. Effect sizes (Cohen’s *d* [ES]) were calculated to determine the magnitude of difference between the 2 groups. Effect sizes were interpreted as small (ES = 0.2-0.5), medium (ES = 0.5-0.8), and large (ES > 0.8) [31]. Post hoc observed power is presented alongside effect sizes and *P* values. All statistical tests were performed in IBM SPSS (version 28). Statistical significance was set at *P* < .05 and adjusted with a Bonferroni correction to account for multiple comparisons. Statistical parametric mapping (SPM) [32] was used to supplement the main statistical analysis to investigate the difference in the time-series kinematic data between patients with a primary and revision THA. To test whether kinematic differences were observed between the 2 groups, we calculated the critical threshold (SPM {t}) at which only *α*% (5%) of smooth random curves would be expected to traverse. The SPM analysis was conducted in MATLAB (The Mathworks, 2021b).

## Results

**Figure 1:**
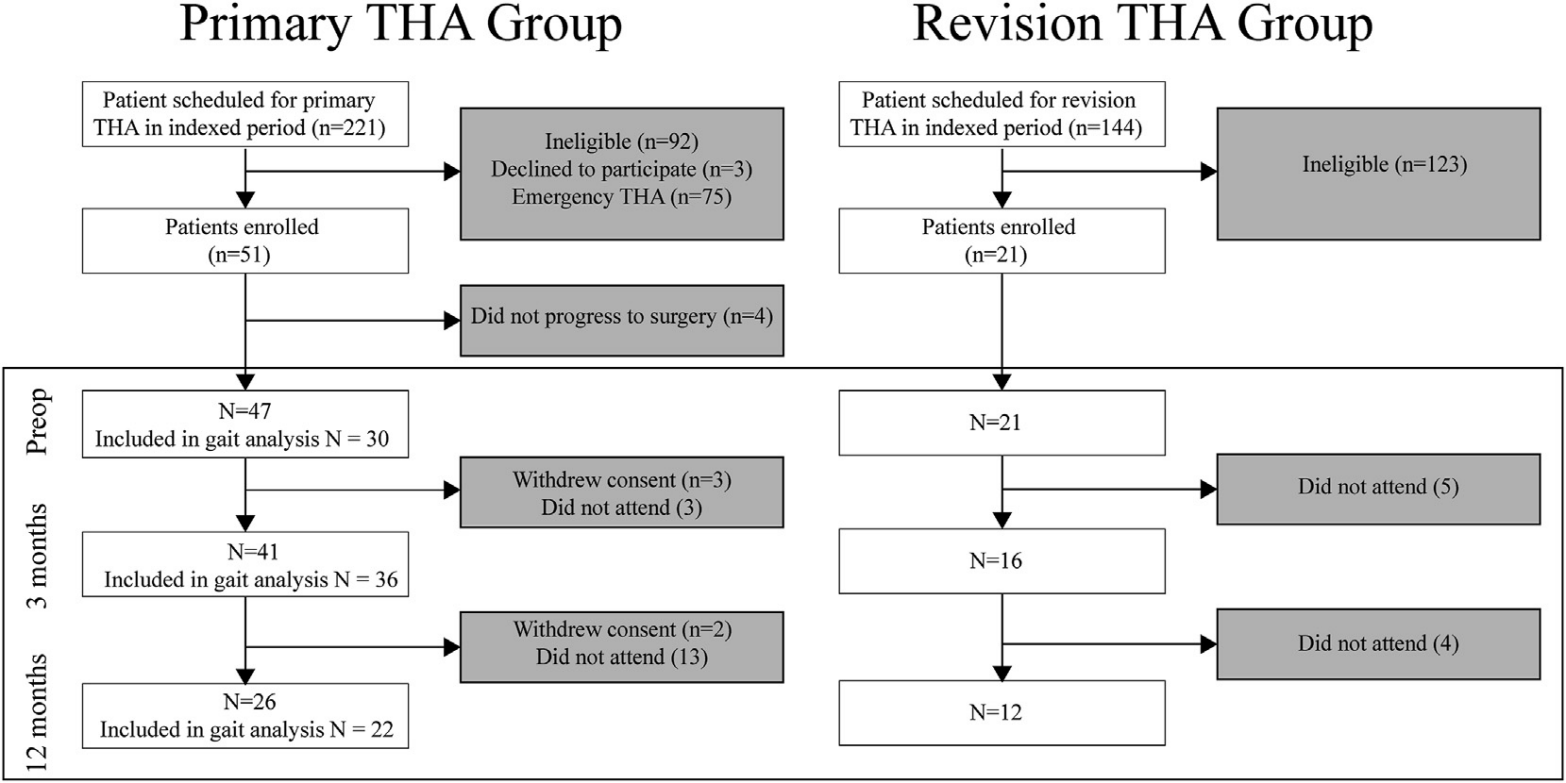
A flowchart describing the recruitment of both groups, eligibility criteria and sample sizes at each time point.

Fifty-one (n = 51) participants undergoing primary THA and 21 (n = 21) undergoing revision THA were recruited. Although no patients were lost to follow-up, several patients did not have all investigations and outcomes recorded at each time point because they withdrew consent for gait analysis or could not attend appointments in person due to remote location or associated pathology (Fig. 1). The final number of patients per group included in the analysis at the individual time points is presented in Figure 1. Table 1 shows the group characteristics at each time point. The univariate analysis of variance did not identify any differences (group∗time) for age (*P* = .765) or body mass index (*P* = .804) between groups. There were no significant differences between the proportion of male and female patients per group at each time point (*P* = 1.000). There were no statistically significant preoperative differences between group for Charlson Comorbidity Index (*P* = .578).

**Table 1:** Mean (95% CI) or median† (range) patient age, preoperative Charlson Comorbidity Index, BMI (kg.m^−2^), and gender ratio (% female) for the primary and revision THA groups.

Surgery type	Age	BMI	CCI†	Sex (M:F [%])
Preoperative				
Primary THA (n = 30)	69.2 (64.2-74.2)	29.3 (27.1-31.6)	5 (0-15)	20:10 (33%)
Revision THA (n = 21)	72.8 (66.8-78.8)	31.8 (29.1-34.5)	3 (0-6)	13:8 (38%)
3 mos				
Primary THA (n = 36)	67.3 (62.7-72.0)	29.0 (26.8-31.1)	-	22:14 (39%)
Revision THA (n = 16)	75.1 (67.7-82.4)	31.2 (27.9-34.5)	-	10:6 (38%)
1 y				
Primary THA (n = 22)	67.9 (62.2-73.5)	27.9 (25.5-30.4)	-	18:8 (31%)
Revision THA (n = 12)	72.1 (62.8-80.4)	32.0 (28.3-35.8)	-	7:5 (42%)

BMI, body mass index; CI, confidence interval; CCI, Charlson Comorbidity Index; F, female; M, male; THA, total hip arthroplasty.

**Figure 2:**
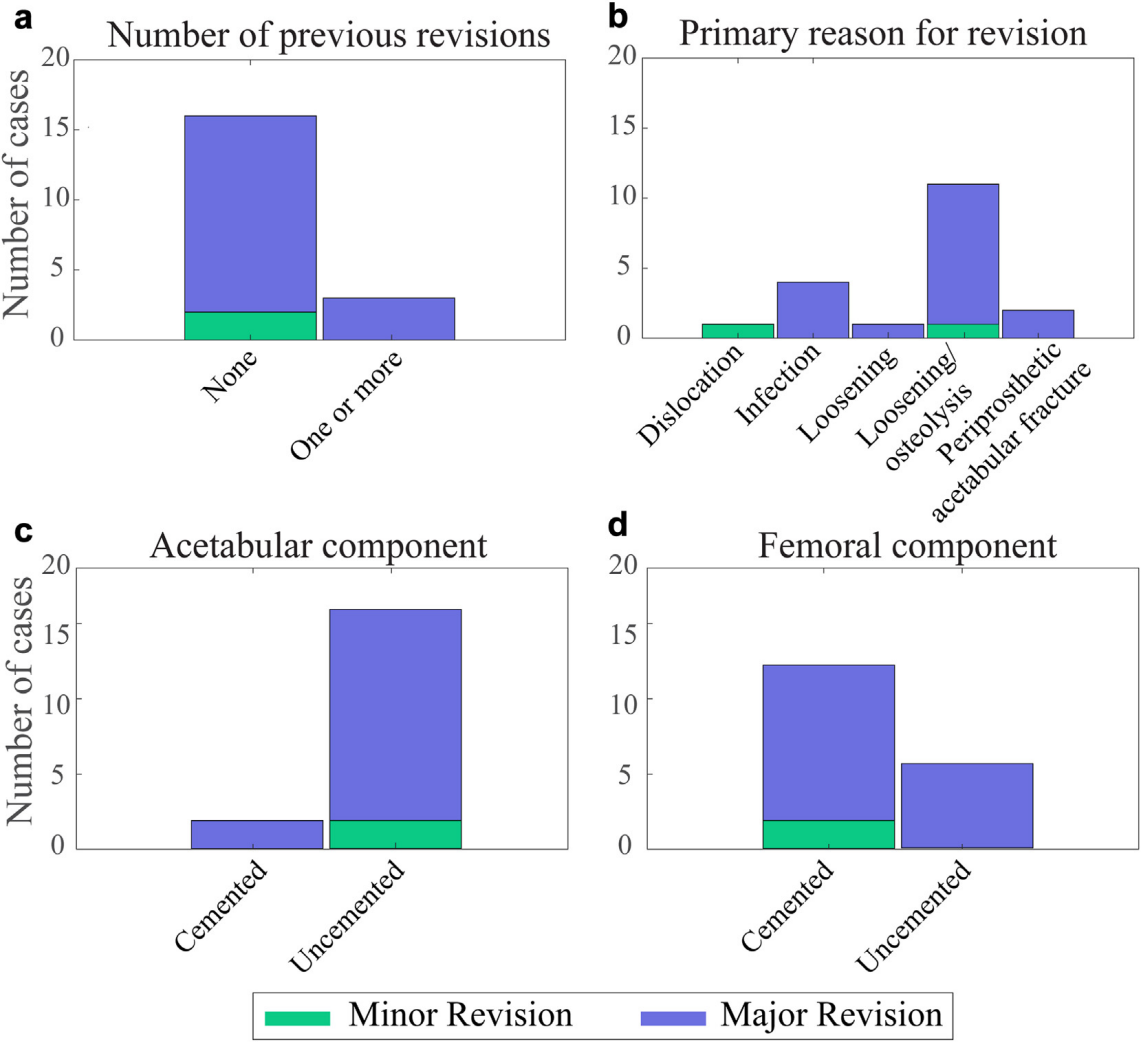
A summary of the number of past revisions (a), the reason for revision (b), and cemented versus uncemented approach taken for the acetabular (c) and femoral (d) component at the time of primary THA. The number of cases is split by what were deemed to be minor and major revisions. THA, total hip arthroplasty.

While all patients undergoing primary THA had the same preoperative diagnosis, for example, hip OA, the patients undergoing revision THA had a heterogeneous set of preoperative characteristics. Figure 2 presents a summary of the reasons for revision, number of previous revisions, and the fixation method used for the femoral and acetabular components.

**Figure 3:**
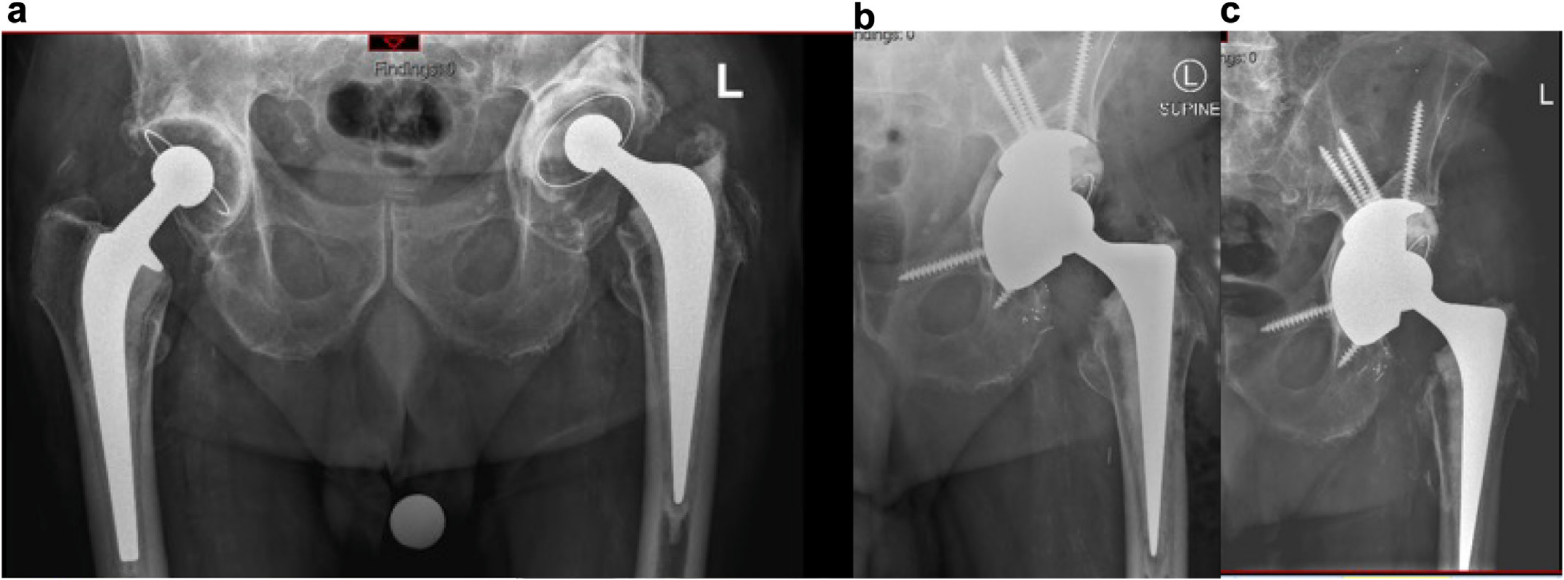
Exemplar radiograph images taken (a) preoperatively, (b) at 3 months postoperative, and (c) at 3 years postoperative from a patient undergoing a revision THA due to loosening and osteolysis. Note that the 3-year radiograph image is shown to demonstrate implant stability. THA, total hip arthroplasty.

In general, the revision THAs were classified as major procedures (90%), in which both femoral and acetabular components, as well as bone composite components, were removed [33] with 4/21 multistage procedures for prosthetic joint infection. An example of a major revision is shown in Figure 3. A detailed summary of preoperative revision THA patient characteristics can be found in Supplementary Table 1. Postoperatively, 4/21 patients in the revision THA group presented with complications within the first postoperative year. In summary, 3 complications were associated with postoperative dislocation, and 1 was due to a femoral periprosthetic fracture.

### Patient-reported outcome measures

**Figure 4:**
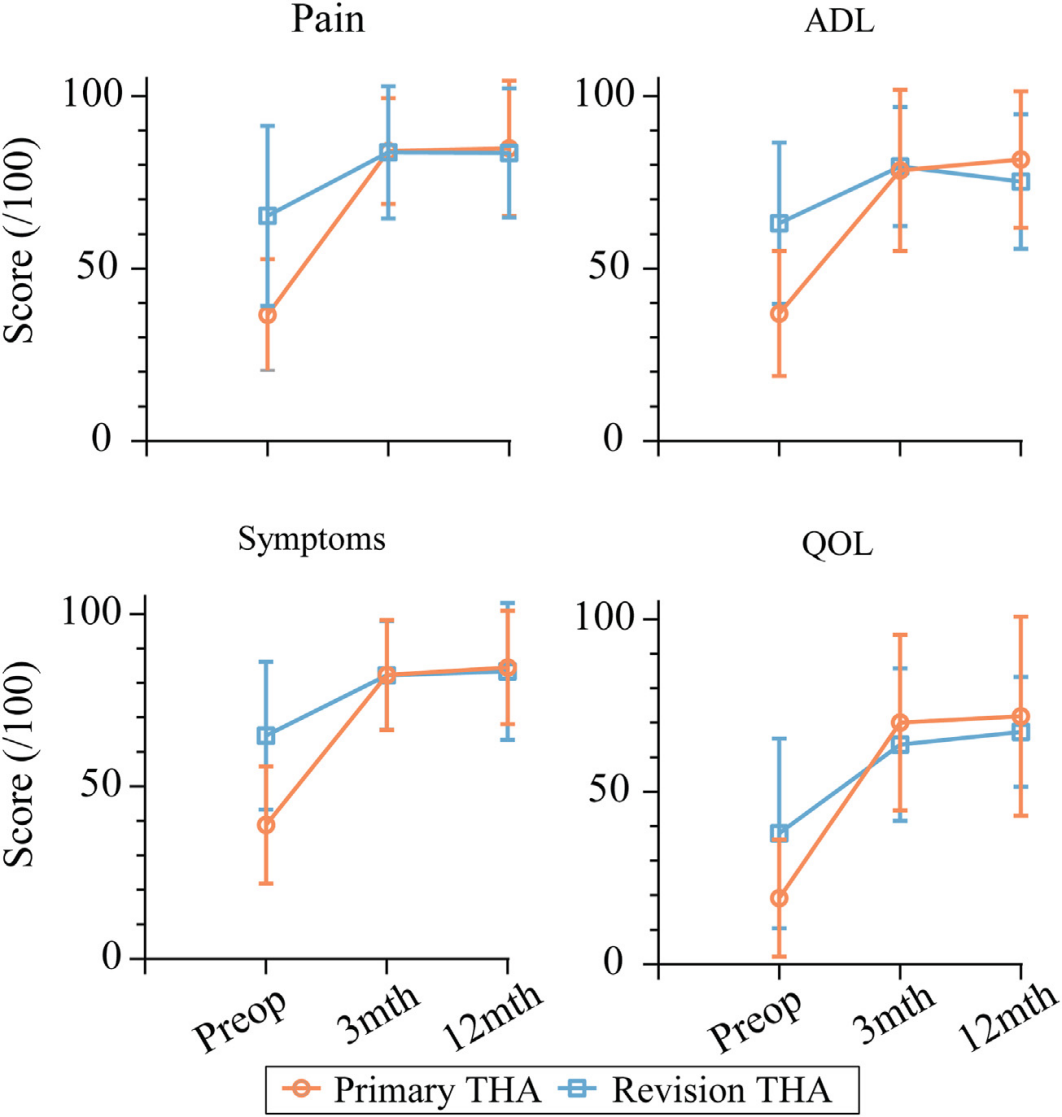
HOOS outcomes for the pain, activities of daily living, symptoms, and quality of life at each time point for the 2 groups of patients. HOOS, Hip Dysfunction and Osteoarthritis Outcome Score.

Preoperatively, patients undergoing primary THA, relative to revision THA, reported significantly worse HOOS scores for pain (mean difference [MD]: 27.4, *P* < .001, ES = 1.5, power = 99%), symptoms (MD: 25.3, *P* < .001, ES = 1.3, power = 99%), activities of daily living (MD: 26.2, *P* < .001, ES = 1.3, power = 99%), and quality of life (MD: 18.7, *P* = .004, ES = 0.9, power = 92%). No significant differences were observed for the 4 HOOS domains at 3 and 12 months between the 2 groups (Fig. 4).

### Gait function

**Figure 5:**
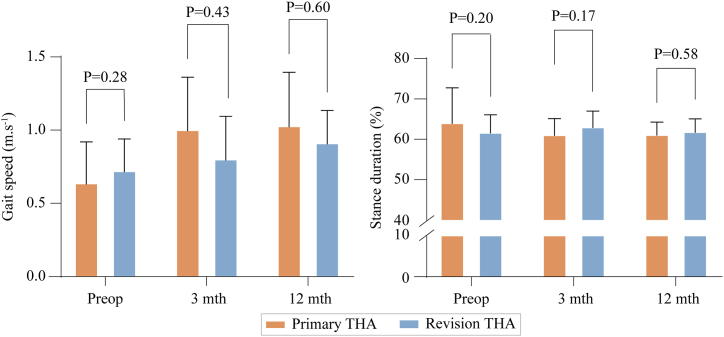
High-level gait metrics (gait speed and stance duration) at each time point for the 2 groups of patients.

No statically significant differences were observed in walking speed between the primary and revision groups at presurgery (Fig. 5) (MD: 0.08 m·s^−1^, ES = 0.30, power = 20%), 3 months (MD: 0.08 m·s^−1^, ES = 0.47, power = 35%), and 12 months (MD: 0.06 m·s^−1^, ES = 0.22, power = 13%) (Fig. 4). There were no statistically significant differences between groups (MD: 2%, 2%, and 1%; ES = 0.28, 0.28, and 0.29, respectively) in the percentage of the gait cycle accounted for by stance at any of the time points (Fig. 5).

For the hip and knee kinematics, none of the differences were statistically significant (*P* > .05) (Table 2). However, given the sample sizes, we complemented our formal statistical analysis with effect sizes (Table 2). We identified medium to large differences (ES > 0.5) in hip extension at 3 and 12 months postoperatively (MD = 9° and 5°, respectively) and hip flexion/extension range of motion (ROM) (MD = 7° and 8°, respectively) (Table 2). There were no statistically significant differences for the proportion of patients with either a Trendelenburg gait or Duchene’s gait at any time point (Table 3). However, Trendelenburg gait was 12% and 13% more common in the revision THA group at 12 and 3 months postoperatively, respectively.

**Table 2:** Mean (95% CI) hip and knee kinematics (degrees) for primary and revision hip THA at the study’s 3 time points.

Surgery type	Time point	Primary THA	Revision THA	*P* value	Cohen’s *d*	Observed power (%)
Peak hip flexion	Preoperative	14 (11-38)	18 (6-43)	.732	0.39	30
	3 mos	16 (7-39)	18 (5-40)	.908	0.12	7
	12 mos	18 (1-37)	16 (2-34)	.823	0.23	12
Peak hip extension	Preoperative	−9 (−35 to 18)	−6 (−31 to 21)	.840	0.24	14
	3 mos	−16 (−41 to 9)	−7 (−32 to 17)	.536	0.64	56
	12 mos	−15 (−36 to 5)	−10 (−30 to 10)	.637	0.54	44
Hip flexion/extension ROM	Preoperative	22 (7-38)	24 (9-39)	.838	0.22	13
	3 mos	32 (16-48)	25 (9-40)	.432	0.89	84
	12 mos	34 (16-51)	26 (9-43)	.437	0.90	85
Peak hip adduction	Preoperative	2 (6-10)	4 (4-12)	.649	0.51	48
	3 mos	5 (4-14)	8 (1-16)	.591	0.63	55
	12 mos	6 (2-13)	6 (1-13)	.957	0.05	5
Peak hip abduction	Preoperative	−5 (−15 to 5)	−5 (−15 to 5)	.979	0.03	5
	3 mos	−4 (−14 to 5)	−2 (−11 to 7)	.583	0.59	50
	12 mos	−5 (−14 to 5)	−4 (−13 to 6)	.844	0.21	11
Hip abduction/adduction ROM	Preoperative	7 (1-13)	10 (4-15)	.517	0.73	78
	3 mos	9 (2-16)	9 (2-16)	.952	0.06	5
	12 mos	10 (3-18)	9 (2-17)	.836	0.24	13
Knee flexion/extension ROM	Preoperative	46 (25-67)	43 (22-63)	.784	0.30	20
	3 mos	52 (32-73)	48 (29-68)	.731	0.35	21
	12 mos	54 (37-72)	52 (35-69)	.789	0.29	16

CI, confidence interval; ROM, range of motion; THA, total hip arthroplasty.

**Table 3:** Proportion of group by time point with either a Trendelenburg or Duchene’s gait.

Surgery type	% Positive Trendelenburg gait	*P* value	% Positive Duchene’s gait	*P* value
Preoperative				
Primary THA	17%	1.000	17%	.734
Revision THA	13%		27%	
3 mos				
Primary THA	31%	.511	3%	1.000
Revision THA	43%		0%	
1 y				
Primary THA	27%	.683	5%	1.000
Revision THA	40%		0%	

THA, total hip arthroplasty.

**Figure 6:**
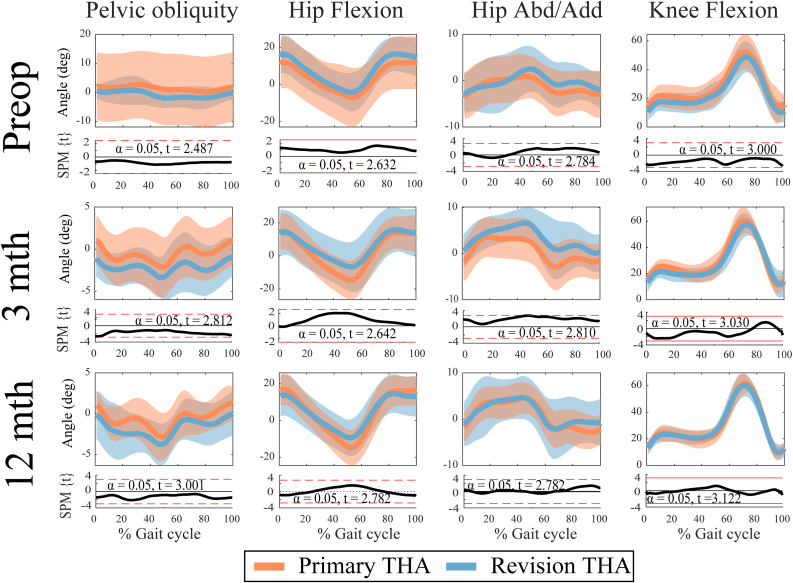
Time-series signals for the pelvic, hip, and knee kinematics at each time point for the 2 groups (top panel for each time point) and SPM output (bottom panel). SPM, statistical parametric mapping.

The SPM analysis revealed no statistically significant differences between the groups for any of the kinematic parameters during the gait cycle at any time point (Fig. 6), although qualitatively hip extension appeared to be reduced during mid-late stance for the revision THA group with respect to the primary THA group at 3 and 12 months postoperatively.

**Supplementary Figure 1:**
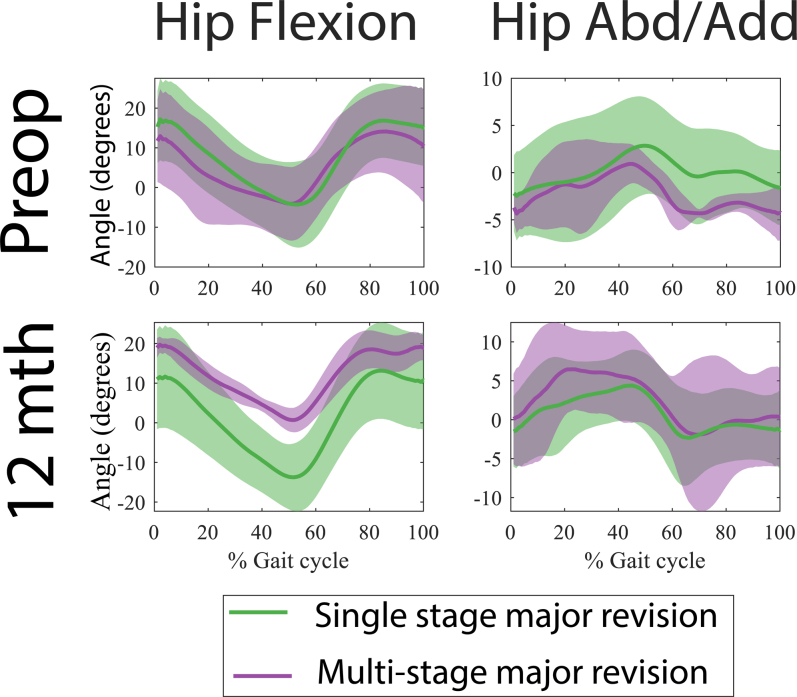


While limited by sample size which forced us to only consider preoperatively and 12 months postoperatively (n = 3 preoperatively, n = 1 at 3 months postoperatively, and n = 3 at 12 months postoperatively), we identified that patients undergoing a multistage revision tended to have similar preoperative hip flexion/extension with respect to those undergoing a single-stage revision (MD of 1° of hip extension at toe off). However, at 12 months postoperatively, those undergoing a single-stage revision for aseptic reasons had on average 14° more hip extension at toe off with respect to patients undergoing a multistage revision for infection (Supplementary Figure 1).

## Discussion

The Adelaide extended posterior surgical approach [15,34] used in the cohort of revision THA patients in this study aimed to protect the hip abductors, which are critical to good postoperative hip function. We hypothesized that by using this surgical approach to protect the glutei, outcomes could be comparable between primary and revision THA patients. Our results show that it may be possible to get similar patient-reported and gait outcomes for primary and revision THA patients. To our knowledge, this is the first study to show that similar gait outcomes are achievable. We identified no between-group differences in PROMs. Patients with end-stage hip OA undergoing primary THA had worse preoperative PROMs when compared to their peers scheduled for revision THA. The postoperative scores for both the primary and revision THA groups were comparable to previous studies reporting 12-month follow-up for the HOOS [35]. Patients in both our primary and revision THA groups showed a plateauing of PROMs between 3 and 12 months postoperatively. This suggests different trajectories to a similar outcome at 12 months postoperatively. The baseline for the 2 groups is different: at primary THA, patients have suffered from years of pain associated with progressively worsening OA; however, at revision THA, the pain associated with end-stage hip OA has been treated with the primary procedure. As pain is a major component of PROMs calculation, this may explain the preoperative differences between the groups and the subsequent trajectories of their recoveries.

Our objective analysis of gait provided better resolution with respect to recovery of physical function. No statistically significant differences were observed for gait outcomes between the 2 groups. Importantly, the outcomes for all spatiotemporal and kinematic variables in this study for the primary THA group reflect previous studies at 3 and 12 months, emphasizing that our findings are not just demonstrative of a poor-performing primary THA cohort [36]. While there were no statistically significant differences in gait outcomes between the 2 groups, some of the effect sizes in hip-specific outcomes were noteworthy suggesting that there may be facets of physical function that could be improved following revision THA using the Adelaide Extended Approach. On average, our results indicate that patients undergoing revision THA tend to take longer to recover hip extension and total hip ROM when compared to patients undergoing primary THA. Patients with a revision THA had 9° and 5° less hip extension at 3 and 12 months postoperatively, respectively. These findings were reinforced by the trajectories seen in the recovery of walking speed following each procedure. While between-group effect sizes for hip flexion-extension were moderate to large, the postoperative differences fall below a previously published minimum clinically important improvement of 13°; although primary THAs attained the minimum clinically important postoperative threshold of 30° for hip flexion/extension ROM, revision THAs failed to achieve minimum clinically important postoperative threshold [37]. Our findings suggest that irrespective of the type of surgery, patients demonstrated similar outcomes at 12 months postoperatively, but the trajectory of recovery differs by procedure. While not statistically significant, the differences in the proportion of patients with a postoperative Trendelenburg gait were noteworthy. Both groups showed an increase in the proportion of patients with a Trendelenburg gait, but this gait pattern was 12%-13% more common in patients following revision THA.

The 2 groups presented in this study reflect the normal mix of patients seen at our public tertiary institution. When we interrogated the data in more detail with regards to the complexity of the surgery, our results were unsurprizing. In this small subgroup analysis of patients, those who underwent a multistage major revision (n = 3) tended to have a substantially smaller ROM and practically no hip extension at toe off at 12 months following surgery. While it is seemingly logical that the complexity and number of previous operations will influence function, there are no objective data confirming this or the level of function a patient may aspire to achieve.

The study has several limitations which must be accounted for when interpreting the results. The sample size for each group was relatively small and the group sizes were unbalanced. We present the observed power as a post hoc test, which highlights the potential issues with our sample size. However, there are significant issues with post hoc observed power tests [38]. Readers are encouraged to focus on the effect sizes and consider *P* values as exploratory. Despite this, the primary THA group is still one of the largest comprehensive datasets we are aware of. Furthermore, the revision THA group was more heterogenous in terms of patient characteristics than the primary THA group with some revisions being minor while others were major complex revisions. This reflects the typical patient mix undergoing revision THA. Both groups demonstrated relatively high levels of Trendelenburg gait. We stress that the classification of this gait pattern is from the motion capture data. These data provide a higher resolution image of the gait patterns when compared to clinical observations. However, we did not directly compare clinical observations and motion capture data in the groups in this study. Furthermore, we cannot definitively state that the original surgical approach was not associated with a postoperative Trendelenburg gait pattern at the time of the primary procedure. Many of the people undergoing revision THA received their primary THA at a different center. Therefore, information on the surgical approach used for the primary procedure for the revision THA group was not always accessible. Finally, we investigated the level walking gait. While this was driven by practical consideration related to safety and physical capabilities of people having revision THA, we acknowledge that other functional tasks such as stair negotiation or sit to stand may be able to highlight deficits that may be unchallenged during level walking.

## Conclusions

This study has demonstrated that it may be possible that revision THA using a gluteal-sparing extended posterior approach can achieve similar outcomes during level walking gait with those of primary THA at 12 months postoperatively. This should stimulate work to make such results generalizable.

## Funding

This work was supported by the National Health and Medical Research Council of Australia (ID: 1126229).

## CRediT authorship contribution statement

**Dominic Thewlis:** Writing – review & editing, Writing – original draft, Visualization, Supervision, Project administration, Methodology, Funding acquisition, Formal analysis, Conceptualization. **Jasvir Bahl:** Writing – review & editing, Writing – original draft, Supervision, Project administration, Formal analysis, Conceptualization. **Hao Wei (Harvey) Chai:** Writing – review & editing, Project administration, Methodology, Formal analysis. **Stuart A. Callary:** Writing – review & editing, Writing – original draft, Supervision, Methodology. **Thomas M. Grace:** Writing – review & editing, Project administration, Methodology, Investigation. **John B. Arnold:** Writing – review & editing, Supervision, Investigation, Conceptualization. **Mark Taylor:** Writing – review & editing, Supervision, Formal analysis, Conceptualization. **Lucian B. Solomn:** Writing – review & editing, Writing – original draft, Supervision, Methodology, Investigation, Formal analysis, Conceptualization.

## Conflicts of interest

Stuart A. Callary received research support from Zimmer Biomet and Corrin as a Principal Investigator and is a board member of the International Radiostereometry Society and the Australian New Zealand Orthopaedic Research Society. All other authors declare no potential conflicts of interest.

For full disclosure statements refer to https://doi.org/10.1016/j.artd.2025.101681.

## Appendix A Supplementary data

Conflict of Interest Statement for All Authors
