# Supplementary material for: Revision Hip Arthroplasty Through a Gluteal-Sparing Extended Posterior Approach May be Able to Achieve Similar Functional Outcomes to Primary Hip Arthroplasty
Source: Arthroplast Today. 2025 Apr 12;33:101681. doi: 10.1016/j.artd.2025.101681 (PMC12017929; doi:10.1016/j.artd.2025.101681)
Supplement: Supplementary Table 1 [file mmc2.docx]

**Supplementary Table:** Detailed description of the revision THA cohort.

| ID | Sex | Age | Nr of revision | Type of revision | Reason | Site | Implant in situ at the time of revision | | | Bone defect reconstructed  (Paprosky) | New implant | | | Complications of the revision |
| --- | --- | --- | --- | --- | --- | --- | --- | --- | --- | --- | --- | --- | --- | --- |
|  |  |  |  |  |  |  | Fixation technique | Name | Company |  |  |  |  |  |
|  |  |  |  |  |  |  |  |  |  |  | Fixation technique | Name | Company |  |
| 1 | F | 75 | 1^st^ | Major | Loosening/  osteolysis | Acetabulum | Uncemented | Vitalock | Howmedica | II B | Uncemented | TM | Zimmer | nil |
|  |  |  |  |  |  | Femur | Uncemented | PCA | Howmedica | - | - | - | - |  |
| 2 | M | 81 | 1^st^ | Minor | Recurrent  dislocation | Acetabulum | Uncemented | Trilogy | Zimmer | - | - | - | - | nil |
|  |  |  |  |  |  | Femur | Cemented | CPT | Zimmer | - | - | - | - |  |
| 3 | M | 78 | 1^st^ | Major | Loosening/  osteolysis | Acetabulum | Cemented | Spectron | Smith & Nephew | II B | Uncemented | Trilogy | Zimmer | nil |
|  |  |  |  |  |  | Femur | Cemented | Spectron | Smith & Nephew | II | Cemented/CWC | CPT | Zimmer |  |
| 4 | M | 87 | 4^th^ | Major | Loosening/  osteolysis | Acetabulum | Uncemented | Trilogy | Zimmer | - | - |  | - | nil |
|  |  |  |  |  |  | Femur | Cemented | CPT | Zimmer | III B | Uncemented | ZMR | Zimmer |  |
| 5 | M | 73 | 1^st^ | Major 2 stages | Infection | Acetabulum | Uncemented | Pinacle | De Puy | II B | Uncemented | Trilogy | Zimmer | nil |
|  |  |  |  |  |  | Femur | Uncemented | Corail | De Puy | II | Cemented/IBG | CPT | Zimmer |  |
| 6 | F | 83 | 1^st^ | Major | Loosening/  osteolysis | Acetabulum |  |  |  | III A | Uncemented | TMARS | Zimmer | Recurrent dislocation – minor revision |
|  |  |  |  |  |  | Femur | Cemented | C Stem | De Puy | III A | Cemented/long stem | CPT | Zimmer |  |
| 7 | M | 50 | 1^st^ | Major 3 stages | Infection | Acetabulum | Uncemented | Trilogy | Zimmer | IIB | Uncemented | TM | Zimmer | 2 dislocations |
|  |  |  |  |  |  | Femur | Cemented | CPT | Zimmer | II | Cemented/IBG | CPT | Zimmer |  |
| 8 | F | 58 | 1^st^ | Major | Loosening/  osteolysis | Acetabulum | Uncemented | Mallory | Biomet | III A | Uncemented | TM | Zimmer | Recurrent dislocation – minor revision |
|  |  |  |  |  |  | Femur | Uncemented | Mallory | Biomet | - | - | - | - |  |
| 9 | M | 71 | 2^nd^ | Major 3 stages | Infection | Acetabulum | Uncemented | PCA | Howmedica | III B | Uncemented | TMARS | Zimmer | Vancouver C periprosthetic fracture - ORIF |
|  |  |  |  |  |  | Femur | Cemented | CPT | Zimmer | II | Cemented/long stem | CPT | Zimmer |  |
| 10 | M | 72 | 1^st^ * | Major | Loosening | Acetabulum | Uncemented | TMARS | Zimmer | II B | Uncemented | TM | Zimmer | nil |
|  |  |  |  |  |  | Femur | Cemented | CPT | Zimmer | - | Cemented/CWC | CPT | Zimmer |  |
| 11 | M | 58 | 1^st^ | Major | Loosening/  osteolysis | Acetabulum | Uncemented | CLS | Protek | III A | Uncemented | TMARS | Zimmer | nil |
|  |  |  |  |  |  | Femur | Uncemented | CLS | Protek | II | Cemented/IBG | CPT | Zimmer |  |
| 12 | M | 74 | 1^st^ | Major | Loosening/  osteolysis | Acetabulum | Uncemented | Mallory | Biomet | II B | Uncemented | TM | Zimmer | nil |
|  |  |  |  |  |  | Femur | Uncemented | Mallory | Biomet | - | - | - | - |  |
| 13 | F | 78 | 2^nd^ | Major | Periprosthetic  acetab fracture | Acetabulum | Uncemented | Spectron | Smith & Nephew | II B | Uncemented | TM | Zimmer | nil |
|  |  |  |  |  |  | Femur | Cemented | Spectron | Smith & Nephew | - | - | - | - |  |
| 14 | F | 81 | 1^st^ | Major | Periprosthetic  acetab fracture | Acetabulum | Uncemented | Spectron | Smith & Nephew | II C | Uncemented | TM | Zimmer | nil |
|  |  |  |  |  |  | Femur | Cemented | Spectron | Smith & Nephew | II | Cemented/IBG | CPT | Zimmer |  |
| 15 | M | 77 | 1^st^ ** | Major 2 stages | Infection | Acetabulum | Uncemented | Vitalock | Howmedica | II B | Uncemented | TM | Zimmer | nil |
|  |  |  |  |  |  | Femur | Cemented | Exeter | Howmedica | II | Cemented/IBG | CPT | Zimmer |  |
| 16 | F | 82 | 1^st^ | Major | Recurrent  dislocation | Acetabulum | Uncemented | CPT | Zimmer | II A | Uncemented | Trilogy | Zimmer | nil |
|  |  |  |  |  |  | Femur | Cemented | Trilogy | Zimmer | - | - | - | - |  |
| 17 | F | 62 | 1^st^ ** | Minor | Loosening | Acetabulum | Uncemented | Trilogy | Zimmer | - | - | - | - | nil |
|  |  |  |  |  |  | Femur | Cemented | CPT | Zimmer | - | Cemented/CWC | CPT | Zimmer |  |
| 18 | M | 87 | 1^st^ | Major | Loosening/  osteolysis | Acetabulum | Cemented | Exeter | Howmedica | III B | Uncemented | TMARS | Zimmer | nil |
|  |  |  |  |  |  | Femur | Cemented | Exeter | Howmedica | - | Cemented/CWC | CPT | Zimmer |  |
| 19 | M | 57 | 1^st^ | Major | Loosening/  osteolysis | Acetabulum | Uncemented | PCA | Howmedica | II B | Uncemented | TM | Zimmer | 1 dislocation |
|  |  |  |  |  |  | Femur | Uncemented | PCA | Howmedica | - | - | - | - |  |
| 20 | M | 59 | 1^st^ | Major | Loosening/  osteolysis | Acetabulum | Uncemented | Quadra | Medacta | II B | Uncemented | TM | Zimmer | nil |
|  |  |  |  |  |  | Femur | Uncemented | Quadra | Medacta | II | Cemented/IBG | CPT | Zimmer |  |
| 21 | F |  | 1^st^ | Major | Loosening/  osteolysis | Acetabulum |  | PCA | Howmedica |  |  |  |  |  |
|  |  |  |  |  |  | Femur |  | PCA | Howmedica |  |  |  |  |  |

* - had primary THR for an acute ABC acetabular fracture

** - had periprosthetic femoral fracture in the past

CWC – cement within cement; IBG – impaction bone grafting
